# Could the novel ‘double-hole’ technique be an alternative for the inflow occlusion method?

**DOI:** 10.5830/CVJA-2016-020

**Published:** 2016

**Authors:** Sahin Bozok, Ilhan Gokhan, Kazdal Izmir,, Ozpak Berkan, Yurekli Ismail, Kestelli Mert, Bayrak Serdar

**Affiliations:** Department of Cardiovascular Surgery, Faculty of Medicine, Recep Tayyip Erdogan University, Training and Research Hospital, Rize, Turkey; Department of Cardiovascular Surgery, Faculty of Medicine, Recep Tayyip Erdogan University, Training and Research Hospital, Rize, Turkey; Department of Anesthesiology and Reanimation, Faculty of Medicine, Recep Tayyip Erdogan University, Training and Research Hospital, Rize, Turkey; Department of Anesthesiology and Reanimation, Faculty of Medicine, Recep Tayyip Erdogan University, Training and Research Hospital, Rize, Turkey; Department of Anesthesiology and Reanimation, Faculty of Medicine, Recep Tayyip Erdogan University, Training and Research Hospital, Rize, Turkey; Department of Anesthesiology and Reanimation, Faculty of Medicine, Recep Tayyip Erdogan University, Training and Research Hospital, Rize, Turkey; Institue of Oncology, Dokuz Eylul University, Izmir, Turkey

**Keywords:** inflow occlusion, foreign body, extraction, double-hole technique, circulation

## Abstract

**Background:**

Inflow occlusion on beating heart and cardiopulmonary bypass techniques have been proposed for the removal of foreign material, such as stents, catheters and mass lesions, from cardiac chambers. However, both techniques are not devoid of disadvantages and complications. In this article, we define an alternative, novel ‘double-hole’ technique, which is based on opening the right atrium without cardiopulmonary bypass .

**Methods:**

Bovine hearts were obtained from a local supermarket. Two purse-string sutures were placed in the right atrium using 2-0 braided, non-absorbable polyester suture material, one close to the auricle, and the other close to the interatrial septum. The guidewire of a haemodialysis catheter was inserted through the superior vena cava into the right atrium and passed all the way through the right ventricle.

**Results:**

We suggest that the double-hole technique may be useful, especially in revision cases with adhesions. Further research should be performed to document the efficacy and safety of this method.

**Conclusion:**

We are aware that further extensive research is necessary to investigate the utility of this novel technique in contemporary cardiovascular surgery. We believe the doublehole technique has the potential to become a safe, practical and effective measure in the future.

## Background

Inflow occlusion on a beating heart (IOBH) is a technique that was used more often in cardiovascular surgery before thecardiopulmonary bypass (CPB) era. Nowadays, this technique is reserved for cases such as pulmonary or aortic valvotomy, cardiac injury, atrial septectomy and extraction of intracardiac thrombus or foreign body.^1^-^3^

CPB can alternatively be used for these operations. Complications may arise due to technical issues, such as tissue injury during cannulation or embolic events. Peri-operative problems arising from the infammatory process caused by extracorporeal circulation signify that CPB is not a technique deviod of complications, in comparison to IOBH.^1^

To eliminate the disadvantages of IOBH and CPB, we have developed a novel technique on a bovine heart. We hope that the ‘double-hole’ technique could provide a safe and effective alternative in the removal of foreign material such as catheters and pacemaker leads.

## Methods

All animal studies were carried out with the approval of the Institutional Animal Care and Use Committee. Bovine hearts were obtained from a local supermarket. Two purse-string sutures were placed in the right atrium using 2-0 braided, non-absorbable polyester suture material (Ticron®, Covidien, Norwalk, CT 06856, USA), one close to the auricle, and the other close to the interatrial septum.

**Fig. 1:**
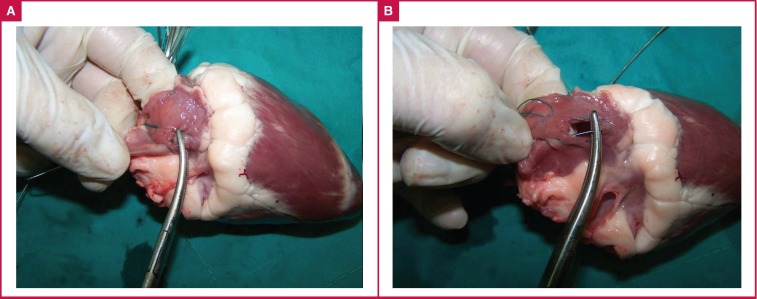
A. Two purse-string sutures are placed, one close to the auricle and the other close to the interatrial septum. The left index finger is inserted into the ventral hole and a closed clamp is inserted into the dorsal hole. B. The clamp is opened inside the right atrium. The clamp is closed after the left index finger pushes the wire between the jaws of the clamp. The wire held by the clamp is extracted.

The guidewire of a haemodialysis catheter was inserted through the superior vena cava into the right atrium and passed all the way through the right ventricle. A stab wound was made within the purse-string sutures and the left index finger wasintroduced into the right atrium through the dilated hole, close to the auricle. In the right hand, a curved haemostatic clamp was introduced through the dilated hole, close to the interatrial septum (Fig. 1A). A guidewire or catheter inside the right atrium was pushed towards the other hole with the tip of the left index finger and caught with the clamp in the other hole, held by the right hand, and extracted (Fig. 1B).

**Fig. 2:**
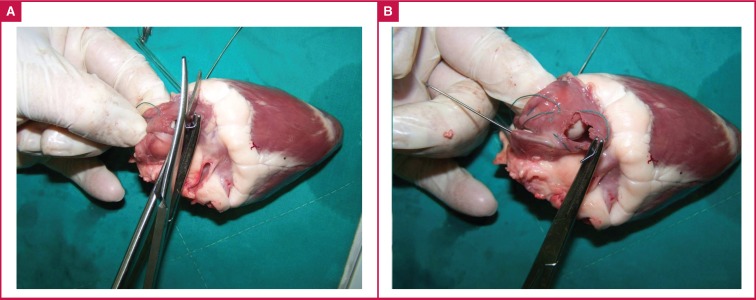
A. The extracted wire is cut into two pieces. B. Removal of the distal part of the wire.

Following visualisation and extraction, the wire was cut into proximal and distal pieces. The proximal piece was extracted (Fig. 2A), and the distal piece was then removed (Fig. 2B). Repetition of this procedure revealed that we were able to retrieve the wire with the clamp every time, and the two pieces of wire were removed, where after the right atrium was closed with snares.

## Discussion

IOBH is a well-known but uncommonly used technique to remove mass lesions and foreign material such as pacemaker leads and catheters from the right atrium.^1^ In this technique, blood flow from the superior and inferior vena cavae to the right atrium is prevented by occlusion with snares, and the right atrium is then opened. This method has significant disadvantages, such as bleeding, hypotension, air embolism, difficulty of surgical exposure, and the necessity to be performed in a short time. Cardiac and neurological complications may occur due to systemic and cerebral malperfusion, particularly in occlusions of more than three minutes.^2^

CPB may be required, particularly in cases with complicated right atrial material. This necessity arises owing to co-morbidities of the patient, extension of the material, and the potential for pulmonary embolism. Studies have demonstrated that the use of CPB is particularly common in cases with co-existence of extracardiac tumours and large, invasive right atrial thrombus.^4^,^6^4-6 Both IOBH and CPB techniques may be used in the extraction of intracardiac pacemaker leads,6 and in tracheal stent implantation.^7^

CPB can alternatively be used for these interventions, but widespread inflammatory response, length of operation and intubation times, and duration of intensive care unit and hospital stays are limitations of the technique.^3^ These limitations become even more apparent in cases with co-morbidities.1 To overcome these disadvantages, we have developed a novel double-hole technique for the removal of foreign material (e.g. catheters, pacemaker leads) in a bovine heart model.

In the IOBH technique, the superior and inferior vena cavae should be free from the surrounding tissue. A polyester tape is placed around each vena cava to provide occlusion of inflow. Complications such as bleeding and air embolisation may be minimised in the double-hole technique since it involves less traumatic and more haemostatic steps such as a pursestring suture, a smaller hole for clamp insertion, and gentler manipulation with the finger.

The technique we describe is easy and practical to perform. While the wire is manipulated with the left index finger, it can easily be grasped repeatedly with the clamp in the right hand. Results of our preliminary report indicate that the double-hole technique could be a safe and effective option for the extraction of pacemaker leads and catheters from the right atrium. We suggest that this technique may be especially useful in revision cases with adhesions. Further research should be performed to document the efficacy and safety of this method.

The main limitation of this experimental study is that the right atria of the bovine heart are much smaller than those of a human heart. Larger atriae may cause more difficulty during surgery. Secondly, the usefulness of this procedure may in fact be limited to wires that are partly trapped in the right atrium, and hence this would include pacer wires and ‘errant’ guidewires. It may not be appropriate for guidewires having left the right atrium and travelled to the right ventricle or pulmonary artery.

## Conclusion

We believe the double-hole technique has the potential to become a safe, practical and effective measure in the future. Further extensive research is necessary to investigate the utility of this novel technique in contemporary cardiovascular surgery. We plan to assess this technique in an in vivo model to corroborate its potential as a less-invasive extraction procedure in future research.
